# Lactic acid level as an outcome predictor in pediatric patients with intussusception in the emergency department

**DOI:** 10.1186/s12887-020-02095-9

**Published:** 2020-04-24

**Authors:** Jeong-Yong Lee, Young-Hoon Byun, Jun-Sung Park, Jong Seung Lee, Jeong-Min Ryu, Seung Jun Choi

**Affiliations:** 1 Department of Pediatrics, Asan Medical Center, University of Ulsan College of Medicine, 88 Olympic-ro 43-gil, Songpa-gu, Seoul, 05505 Republic of Korea; 2 Department of Emergency Medicine, Asan Medical Center, University of Ulsan College of Medicine, 88 Olympic-ro 43-gil, Songpa-gu, Seoul, 05505 Republic of Korea

**Keywords:** Child, Emergency service hospital, Intussusception, Lactic acid, Prognosis

## Abstract

**Background:**

Intussusception decreases blood flow to the bowel, and tissue hypoperfusion results in increased lactic acid levels. We aimed to determine whether lactic acid levels are associated with pediatric intussusception outcomes.

**Methods:**

The electronic medical records of our emergency department pediatric patients diagnosed with intussusception, between January 2015 and October 2018, were reviewed. An outcome was considered poor when intussusception recurred within 48 h of reduction or when surgical reduction was required due to air enema failure.

**Results:**

A total of 249 patients were included in the study, including 39 who experienced intussusception recurrence and 11 who required surgical reductions; hence, 50 patients were included in the poor outcome group. The poor and good outcome groups showed significant differences in their respective blood gas analyses for pH (7.39 vs. 7.41, *P* = .001), lactic acid (1.70 vs. 1.30 mmol/L, *P* < .001), and bicarbonate (20.70 vs. 21.80 mmol/L, *P* = .036). Multivariable logistic regression analyses showed that pH and lactic acid levels were the two factors significantly associated with poor outcomes. When the lactic acid level cutoff values were ≥ 1.5, ≥2.0, ≥2.5, and ≥ 3.0 mmol/L, the positive predictive values for poor outcomes were 30.0, 34.6, 50.0, and 88.9%, respectively.

**Conclusion:**

Lactic acid levels affect outcomes in pediatric patients with intussusception; higher lactic acid levels are associated with higher positive predictive values for poor outcomes.

## Background

Intussusception, an abdominal emergency, is one of the most frequent causes of bowel obstruction in the pediatric population [1, 2]. Its treatment involves reduction, using an air or barium enema, or, in some cases, surgical reduction [3, 4]. If intussusception is not relieved, the bowel vascular supply becomes compromised, causing intestinal ischemia progression and a risk of perforation. Therefore, identifying the conditions that increase the risk of a poor outcome (e.g., recurrence or difficult-to-relieve cases) is important. Previous studies have described the risk factors for recurrent intussusception, including age, presence of pathological leading points, and symptom duration [5–8]. However, the association between specific laboratory findings and outcomes has not been investigated.

Elevated lactic acid levels occur secondary to tissue hypoperfusion/hypoxia or to causes unrelated to tissue hypoxia. Hypoperfusion-driven cases include all forms of shock, post-cardiac arrest, and regional ischemia [9]. Interestingly, reversible or irreversible intestinal ischemia develops following intussusception, potentially causing elevated lactic acid levels. In this study, we evaluated the association between lactic acid levels at the time of intussusception diagnosis and outcomes.

## Methods

This retrospective study analyzed data retrieved from the electronic medical records of pediatric patients diagnosed with intussusception, between January 2015 and October 2018 in our emergency department (ED). Patients diagnosed with the ileo-colic type of intussusception, which is primarily treated using non-surgical reduction, were included. An outcome was defined as poor when (1) nonsurgical reduction failed and the patient required surgery, (2) intussusception recurred during the post-procedural observation period, in the ED, following nonsurgical reduction, or (3) the patient revisited the ED due to recurrence within 48 h of nonsurgical reduction. Patients were excluded if leading points that increase the risk of recurrence, like polyps or a diverticulum, were identified during their abdominal ultrasound examination; the patient was discharged against the physician’s advice; or when the initial blood gas analysis results were missing.

The collected patient data included demographic data, symptoms at initial presentation, time between symptom onset and hospital visit, time between symptom onset and nonsurgical reduction, number of nonsurgical reduction attempts, whether or not surgical reduction was performed, intussusception recurrence, and venous blood gas analysis (VBGA) results (pH, lactic acid, and bicarbonate levels).

Following our institutional protocol, when intussusception was clinically suspected, the pediatric emergency physician performed a point-of-care ultrasound (POCUS) examination. If the POCUS results led to an intussusception diagnosis, rapid intravenous hydration was performed and a radiologist was simultaneously consulted to perform a fluoroscopy-guided air enema, as soon as possible. The degree of acid-base imbalance and lactic acid levels were evaluated prior to intravenous hydration, using a blood gas analyzer. If the air enema was successful, the patient remained in the ED without orally consuming anything for 6 h. Thereafter, possible recurrence was ruled out using either plain radiography or POCUS. In the absence of recurrence, the patient was started on a liquid diet and was discharged, after confirming the absence of emesis and abdominal pain. If the air enema failed to relieve the intussusception, or when there was recurrence during the post-procedural observation period, another enema was attempted or pediatric surgeons were consulted regarding surgical intervention.

All statistical analyses were performed using IBM SPSS Statistics for Windows, version 21.0 (IBM Corp., Armonk, New York). The included patients were divided into the poor and good outcome groups and compared for differences in demographic data and clinical parameters using Wilcoxon rank-sum tests, chi-square tests, or Fisher’s exact tests, as appropriate. Risk factor analysis for the prediction of poor outcomes was conducted using multivariable logistic regression model. Reliability values (sensitivity and specificity) and predictive values (positive predictive value and negative predictive value) for poor outcomes were analyzed using different lactic acid cutoff levels. For all analyses, *P* values < .05 were considered statistically significant.

## Results

A total of 296 patients with ileo-colic intussusception visited the ED during the study period. Of these, 249 patients were included in the analysis, after excluding patients with incomplete data (no VBGA results or missing pH, lactic acid, or bicarbonate measurements, *n* = 39), leading points (*n* = 5), or who had been transferred to other hospitals (n = 3). The median age of the included patients was 1.8 years, and 63.9% were male. The clinical and demographic characteristics of patients are summarized in Table 1. Fifty patients were included in the poor outcome group, including 11 who underwent surgical reductions due to air enema failures, 20 who experienced recurrence during the post-procedural observation period, and 19 who otherwise suffered recurrence.

**Table 1:** Clinical and demographic characteristics of patients (*n* = 249)

Characteristic	Value
Age, years	1.8 [1.1, 2.7]
Male, n (%)	159 (63.9)
Symptom^a^	
Abdominal pain/irritability, n (%)	207 (83.1)
Vomiting, n (%)	96 (38.6)
Blood-tinged stool, n (%)	41 (16.5)
Time between symptom onset and hospital visit (hours)	7.5 [3.0, 24.0]
Time between symptom onset and air reduction (hours)	9.0 [5.0, 25.0]
Surgery, n (%)	11 (4.4)

Results are presented as medians [interquartile range] and numbers (%)

^a^ Multiple symptoms were listed if more than one was described by the patient or parents

The clinical parameters (age, sex, symptoms at the initial visit, time between symptom onset and hospital visit, and time between symptom onset and nonsurgical reduction) were similar between the good and poor outcome groups. However, pH, lactic acid, and bicarbonate levels were significantly different between the two groups (Table 2). Among these three possible risk factors, the multivariable logistic regression analysis indicated that only pH and lactic acid levels were significantly different between the two groups (Table 3). When the predictive values for poor outcomes were evaluated using different lactic acid cutoff levels, the cutoff values of 1.5 mmol/L, 2.0 mmol/L, 2.5 mmol/L, and 3.0 mmol/L yielded positive predictive values of 30.0, 34.6, 50.0, and 88.9%, respectively; the respective negative predictive values were 87.8, 83.8, 82.5, and 82.5% (Table 4).

**Table 2:** Clinical parameters compared according to outcome

	Poor outcome (*n* = 50)	Good outcome (*n* = 199)	*P*
Age, years	1.92 [1.17, 3.10]	1.83 [1.08, 2.67]	.418
Male, n (%)	35 (70.0)	124 (62.3)	.329
Symptom^a^			
Abdominal pain/irritability, n (%)	43 (86.0)	164 (82.4)	.762
Vomiting, n (%)	21 (42.0)	75 (37.7)	.627
Blood-tinged stool, n (%)	7 (14.0)	34 (17.1)	.675
Time between symptom onset and hospital visit (hours)	8.50 [2.00, 38.00]	7.00 [3.00, 24.00]	.459
Time between symptom onset and air reduction (hours)	10.50 [4.00, 39.00]	9.00 [5.00, 25.00]	.425
Venous blood gas analysis			
pH	7.39 [7.36, 7.43]	7.41 [7.38, 7.45]	.001
Lactic acid (mmol/L)	1.70 [1.30, 2.33]	1.30 [1.08, 1.70]	< .001
Bicarbonate (mmol/L)	20.70 [19.25, 22.65]	21.80 [20.00, 23.50]	.036

Results are presented as medians [interquartile range] and numbers (%)

^a^ Multiple symptoms were listed if more than one was described by the patient or parents

**Table 3:** Multivariable logistic regression analysis for the prediction of poor outcomes

Variables	aOR (95% CI)	*P*
Age	1.026 (1.003–1.049)	.024
Time between symptom onset and hospital visit (hours)	0.868 (0.556–1.354)	.531
Time between symptom onset and air reduction (hours)	1.163 (0.745–1.814)	.507
pH	0.000 (0.000–0.033)	.003
Lactic acid (mmol/L)	3.066 (1.694–5.551)	< .001
Bicarbonate (mmol/L)	0.889 (0.759–1.042)	.148

*aOR* Adjusted odds ratio, *CI* Confidence interval

**Table 4:** Predictive values associated with lactic acid levels

Lactic acid level (mmol/L, n)	Sensitivity (%, n)	Specificity (%, n)	PPV (%, n)	NPV (%, n)	OR (95% CI)	*P*
≥1.5 (110/249)	66.0 (33/50)	61.3 (122/199)	30.0 (33/110)	87.8 (122/139)	3.076 (1.604–5.897)	.001
≥2.0 (52/249)	36.0 (18/50)	82.9 (165/199)	34.6 (18/52)	83.8 (165/197)	2.730 (1.376–5.417)	.006
≥2.5 (20/249)	20.0 (10/50)	95.0 (189/199)	50.0 (10/20)	82.5 (189/229)	4.725 (1.845–12.103)	.002
≥3.0 (9/249)	16.0 (8/50)	99.5 (198/199)	88.9 (8/9)	82.5 (198/240)	37.714 (4.594–309.634)	< .001

*PPV* Positive predictive value, *NPV* Negative predictive value, *OR* Odds ratio, *CI* Confidence interval

## Discussion

From our study results, we found out that lower pH values and higher lactic acid levels were associated with greater likelihoods of intussusception recurrence. Notably, although the difference in the numerical pH values between the groups was statistically significant, the values for both groups were within the normal range. Thus, the lactic acid level is probably the only clinically meaningful parameter associated with poor outcomes in this setting. Using different lactic acid cutoff levels (≥1.5, ≥2.0, ≥2.5, and ≥ 3.0 mmol/L), all of the determined negative predictive values were high but the positive predictive values increased as the cutoff values increased. In particular, the positive predictive value for a poor outcome increased from 50 to 88.9% when the cutoff value was increased from ≥2.5 mmol/L to ≥3.0 mmol/L.

In previous studies, patient lactic acid levels were reported to be poor parameters for diagnosing intussusception [10]. Hence, the diagnostic method of choice is ultrasonography [11]. However, a recent study showed that POCUS, performed by emergency physicians, had a similar diagnostic accuracy as radiologist-performed ultrasound [12]. POCUS is widely used in many institutions, including ours, because it can be promptly performed in the ED.

Based on our study results, lactic acid levels were found to be potential predictors of poor outcomes in pediatric intussusception patients. Although there is no clear definition for an “elevated” lactic acid level, most previous studies report levels of 2.0 or 2.5 mmol/L to indicate elevation [13]. In our study, when a 2.5-mmol/L cutoff was used, 50% of the patients presented poor outcomes; when a 3.0-mmol/L cutoff was used, the positive predictive value increased to 88.9%.

In patients presenting with abdominal pain associated with suspicious mesenteric ischemia, lactic acid level measurements can help guide further diagnostic workups [14]. According to Lange et al., elevated lactic acid levels showed a 96% sensitivity and 38% specificity for indicating mesenteric ischemia [15]. Furthermore, serum lactic acid levels > 2 mmol/L may be associated with irreversible intestinal ischemia [16]. In our study, none of the 11 patients who underwent surgical reduction required resection due to bowel ischemia. Xian-Ming et al. concluded that the median time between symptom onset and operative treatment for intussusception was longer in patients who lost intestinal viability (42 h) than for those who did not (19 h) [17]. In our study, the median time between symptom onset and the procedure was 9 h, which was shorter than that reported by Xian-Ming et al., suggesting that intestinal viability had been preserved.

This study has some limitations. The retrospective nature of the study is the first limitation. Secondly, due to our institutional protocol, only VBGA were performed for most of the patients. Thus, we were unable to analyze the presence of leukocytosis or hemoconcentration, which can be observed in patients with acute mesentery ischemia. Nevertheless, elevated lactic acid levels are early signs of tissue hypoxia and can be used as markers for mesenteric ischemia that are more specific than C-reactive protein levels or leukocyte counts [18, 19]. Future research should include a large, prospectively registered population with additional laboratory findings available for analysis. Furthermore, an evaluation of the association between serial lactic acid levels and outcomes is warranted.

To the best of our knowledge, this is the first study to elucidate an association between intussusception outcomes and laboratory data. Although only a few variables were analyzed, we successfully elucidated an association between the increased risk of poor outcome and increased lactic acid levels; theoretically, lactic acid is a marker of tissue hypoperfusion.

## Conclusion

Elevated lactic acid levels are associated with poor outcomes in pediatric patients with intussusception. In particular, lactic acid levels ≥2.5 mmol/L imply a greater risk for failed nonsurgical reduction or intussusception recurrence, warranting preparedness for alternative treatment strategies.

## Data Availability

The datasets used and analyzed during the current study are not publicly available due to their containing information that could compromise the privacy of study participants, but are available from the corresponding author on reasonable request.

## Abbreviations

ED: Emergency department
POCUS: Point-of-care ultrasound
VBGA: Venous blood gas analysis
